# Is there an association between molar-incisor hypomineralization and carious lesions in seven to ten-year-old Brazilian schoolchildren? A cross-sectional study

**DOI:** 10.1590/1678-7757-2024-0538

**Published:** 2025-06-27

**Authors:** Karina Kendelhy SANTOS, Patrícia Gomes FONSECA, Maria Letícia RAMOS-JORGE, Maria Eliza da Consolação SOARES, Izabella Barbosa FERNANDES

**Affiliations:** 1 Universidade Federal dos Vales do Jequitinhonha e Mucuri Faculdade de Ciências Biológicas e da Saúde Departamento de Odontopediatria Diamantina Brasil Universidade Federal dos Vales do Jequitinhonha e Mucuri, Faculdade de Ciências Biológicas e da Saúde, Departamento de Odontopediatria, Diamantina, Brasil.; 2 Universidade Federal de Minas Gerais Faculdade de Odontologia Departamento de Odontologia Belo Horizonte Brasil Universidade Federal de Minas Gerais, Faculdade de Odontologia, Departamento de Odontologia, Belo Horizonte, Brasil.; 3 Universidade Federal de Juiz de Fora Instituto de Ciências da Vida Departamento de Odontologia Governador Valadares MG Brasil Universidade Federal de Juiz de Fora, Instituto de Ciências da Vida, Departamento de Odontologia, Governador Valadares, MG, Brasil.

**Keywords:** Molar-Incisor hypomineralization, Dental caries, Child, Epidemiology, Enamel development defects

## Abstract

**Objective:**

To evaluate whether the presence of molar-incisor hypomineralization (MIH) is associated with a greater number of decayed teeth in schoolchildren.

**Methodology:**

A cross-sectional study was conducted with a random sample of 347 children aged from seven to ten years in the Brazilian city of Diamantina. The International Caries Detection and Assessment System (ICDAS) criteria were used to determine the number of teeth with moderate/extensive caries (ICDAS codes 3-6). MIH was assessed according to the criteria of the European Academy of Paediatric Dentistry (EAPD). Sociodemographic data and data regarding children's habits were obtained via a questionnaire sent to parents/guardians. Descriptive analyses, Mann Whitney and Kruskal Wallis tests, and Poisson regression were performed.

**Results:**

The prevalence of MIH was 20.5% and that of moderate/extensive caries was 39.2%. The mean number of teeth with moderate/extensive caries was 1.80 (SD±2.67). The mean number of decayed permanent teeth was 0.69 (SD=1.21), and the mean number of decayed primary teeth was 1.11 (SD=1.89). The number of teeth with moderate/extensive caries was associated with the presence of MIH in children (PR=1.45; 95% CI=1.03-2.04; p=0.031). Furthermore, the number of teeth with moderate/extensive caries was associated with lower monthly family income, high frequency of sugar consumption, and visible plaque (p<0.05).

**Conclusion:**

The presence of MIH is associated with a greater number of decayed teeth in schoolchildren.

**Clinical relevance:**

This study suggests that children with MIH are at higher risk of developing caries, emphasizing the importance of specific preventive care and early treatments for this condition. This can influence clinical practices, public health policies, and parental education.

## Graphical abstract

**Figure:**
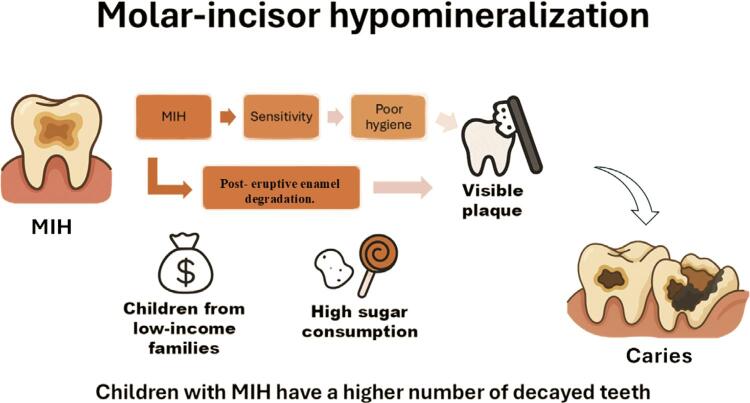

## Introduction

Dental caries is a dynamic multifactorial disease mediated by biofilm, modulated by diet, and characterized by mineral loss from dental hard tissues.^1^ Despite prevention developments, caries is still one of the most prevalent chronic diseases in children worldwide, with an approximate prevalence of 46.2% in primary teeth and 53.8% in permanent teeth.^2^

Among the factors associated with the occurrence of caries, socioeconomic aspects, high levels of mutans streptococci, frequent consumption of sugary foods, the presence of visible plaque, and enamel defects such as molar-incisor hypomineralization (MIH) stand out.^3,6-7^ MIH is a defect in tooth enamel development that affects from one to four first permanent molars, and it is often associated with permanent incisors.^8^ Studies show there is a global prevalence of MIH of around 14.2%.^9^ Clinically, teeth affected by MIH show demarcated opacities, varying in color and size, frequently associated with post-eruptive rupture, restorations and/or atypical caries lesions, and atypical extractions of the first permanent molars.^8^ Furthermore, such teeth have a hypomineralized enamel substructure and may be hypersensitive, which impairs hygiene and contributes to the stagnation of dental plaque, which can result in greater development and progression of caries.^8^ Studies have suggested children with MIH have a higher prevalence of caries,^6^ and new well-designed studies with appropriate methodology and a representative sample have been encouraged.^4-7^ This association plausibility involves a greater susceptibility of hypomineralized teeth to the development and progression of carious lesions due to the reduction in hardness and in the elasticity modulus of hypomineralized enamel, which leads to post-eruptive enamel degradation that favors the accumulation of biofilm due to enamel irregularity.^4^ Morphological and chemical characteristics of MIH defects also contribute to greater local acid solubility, permeability, and faster penetration of oral bacteria, thus making affected teeth more susceptible to caries.^6,7,10^ Another explanation regards the sensitivity in hypomineralized teeth, which hinders cleaning and the removal of biofilm, increasing the susceptibility of developing caries, not only in teeth with MIH but also in adjacent teeth.^4,5^

Understanding whether the presence of MIH can be a risk factor for caries is essential to guide individualized clinical approaches and to the development of preventive public interventions in oral health by dentists and health agencies according to the demand for each population and context. Therefore, this study aimed to evaluate whether the presence of MIH is associated with a higher number of carious teeth in schoolchildren from seven to ten years old in the city of Diamantina, Minas Gerais, Brazil. The null hypothesis is that the number of decayed teeth is not associated with the presence of MIH in schoolchildren.

## Methodology

### Ethical aspects

This study was approved by the Research Ethics Committee of UFVJM (Federal University of Vales do Jequitinhonha and Mucuri) (Statement 5.915.760). The parents or guardians were informed about the aim of the study and signed the Informed Consent Form (ICF) agreeing with their participation. Patients who needed dental treatment were referred to the UFVJM postgraduate clinic. This study followed the guidelines of “Strengthening the Reporting of Observational Studies in Epidemiology”^11^ for cross-sectional studies.

### Population and study design

A population-based cross-sectional study was conducted in public and private schools in the urban area of Diamantina/Brazil between February and July 2023. According to the latest national demographic census, Diamantina has a population of 45,880 inhabitants, with a schooling rate of 97.8%^12^ for children aged from six to 14. The GINI index of the municipality is 0.57.^13^ The city has 12 schools for this age group in the urban area, eight public and four private, with approximately 1,685 enrollments in elementary education.

Schoolchildren aged from seven to ten years whose first four permanent molars had already erupted were included in the research, accompanied by their parents/guardians. The children should be regularly enrolled in school to be eligible for the study. Children who refused to participate in the research and those with neuropsychomotor disabilities and/or cognitive disorders reported by their parents or guardians were excluded.

This study is part of a larger project in which the prevalence of MIH among children aged from seven to ten years was evaluated. For the sample size calculation, OpenEpi version 3 (www.openepi.com2.5) was used, with an open-source PowerMean calculator. A MIH prevalence of 25% (pilot study data) was adopted, with a confidence level of 95%, a standard error of 5%, and a correction factor of 1.2 (design effect), resulting in a minimum initial sample of 331 children. An additional 20% was added to the sample to account for potential dropouts. Thus, the total calculated sample was 398 children. There was a secondary aim of the main project, the obtained sample (n=349) was used and the sample power was then calculated. OpenEpi, version 3, was again used to calculate the power of the sample. Mean, standard deviation, and sample size data in each group (children with and without MIH) were used (Table 1).

**Table 1:** Distribution of the mean number of teeth with moderate/extensive caries according to the independent variables.

Variables	n (%)	Teeth with moderate/extensive caries		*p*
		Median (Minimum-Maximum)	Mean (SD)	
**Sex of the child**				
Female	175 (50.4)	0.00 (0-14)	1.66 (2.66)	0.130*
Male	172 (49.6)	1.00 (0-14)	1.95 (2.67)	
**Child's age**				
7 years	50 (14.4)	0.00 (0-12)	2.18 (3.07)	0.574**
8 years	98 (28.2)	1.00 (0-14)	2.03 (2.77)	
9 years	112 (32.3)	0.00 (0-14)	1.70 (2.66)	
10 years	87 (25.1)	0.00 (0-10)	1.47 (2.27)	
**Caregiver's education**				
Higher education	104 (30.8)	0.00 (0-8)	1.11 (1.96)	0.006**
High school	178 (52.7)	0.00 (0-14)	2.03 (2.85)	
Basic education	56 (16.6)	1.00 (0-14)	2.13 (2.96)	
**Monthly family income**				
>3 minimum wages	68 (19.8)	0.00 (0-7)	0.79 (1.54)	<0.001**
1 to 3 salaries	177 (51.0)	0.00 (0-14)	1.69 (2.70)	
<1 minimum wage	98 (28.2)	2.00 (0-12)	2.68 (2.95)	
**Income dependents**				
≤3 people	127 (36.6)	0.00 (0-12)	1.59 (2.49)	0.441*
>3 people	220 (63.4)	0.00 (0-14)	1.91 (2.75)	
**Sugar consumption**				
<2 times a day	236 (68.0)	0.00 (0-11)	1.27 (2.24)	<0.001*
≥2 times a day	111 (32.0)	2.00 (0-14)	2.92 (3.13)	
Tooth brushing				
≥2 times a day	291 (83.9)	0.00 (0-14)	1.62 (2.45)	0.005*
<2 times a day	56 (16.1)	1.00 (0-14)	2.59 (3.54)	
**Visible Plaque Index**				
<20%	190 (54.8)	0.00 (0-14)	0.82 (1.76)	<0.001*
≥20%	157 (45.2)	2.50 (0-14)	2.99 (3.07)	
**MIH**				
Absence	276 (79.5)	0.00 (0-14)	1.61 (2.47)	0.010*
Presence	71(20.5)	1.00 (0-14)	2.51 (3.23)	

* Mann-Whitney Test; ** Kruskal Wallis Test.

The proportional stratified random sampling technique (public and private schools) was conducted. The eligible children were invited to participate in the study. The draw was performed online using the website sorteador.com, in which random numbers were assigned to each school in Diamantina until the sample size was completed. Six schools were selected, in which four were state public schools and two were private schools.

### Training, calibration, and pilot study

Before data collection, two researchers underwent training and calibration, initially receiving theoretical-practical training on intraoral clinical diagnostic criteria. The training was coordinated by a seasoned researcher and included theoretical explanations and projection of images of different clinical conditions, such as tooth decay and defects in enamel development.

After the training, a calibration was conducted in which the examiners performed a clinical dental examination on 30 children aged from seven to ten years who attended the Pediatric Dentistry clinics at UFVJM. After 15 days, the clinical examination was repeated to verify intra-examiner agreement. This sample was also examined by an experienced reference researcher. Intra-examiner and inter-examiner agreement was assessed using the Kappa coefficient, which showed a minimum value of 80%.

A pilot study was conducted to test the methodology and provide data for sample calculation, following all stages of data collection (intraoral clinical examination and questionnaire). This study was conducted with 31 children (10% of the sample) who were included in the main study, as no changes in methods were necessary.

### Data collection

Initially, the project was shown to the regional board of education, and after being approved, visits to the selected schools were made to show the study and invite students to participate. After acceptance by the educational institution, an envelope containing the research presentation letter, the informed consent form, and a questionnaire were sent to the parents and guardians. They had seven days to agree to participate and return the envelope with the completed and signed documents. Intraoral clinical examinations were conducted in a private, bright, and airy room at the school in children whose caregivers agreed to participate.

### Non-clinical data collection

To collect non-clinical data, a questionnaire was sent to parents/guardians on demographic and socioeconomic characteristics of the family, sex (Female or Male), child’s age (in years), primary caregiver’s education (Higher education (≥12 years of study), High school (nine to 11 years of study), or Basic education (≤8 years of study), monthly family income (based on the Brazilian minimum monthly wage =R$1,320.00 / US$ 264.68 in 2023 (>3 minimum wages, from one to three minimum wages or <1 minimum wage)), number of dependents on the monthly family income (≤3 people or >3 people), frequency of daily tooth brushing (two or more times a day, only once a day, no brushing, or rarely brushing) and how often the child usually has sugary snacks (food or drinks) per day (≤2 times a day, >2 times a day according to Peres, et al.^14^ (2017).

### Clinical data collection

Intraoral examinations were performed with the child sitting in a chair under artificial light (headlamp) and with the aid of a mouth mirror and a spherical probe. The teeth were examined in relative isolation using cotton rolls. All procedures were conducted according to biosafety standards.

Initially, the visible plaque index was visually assessed on the 12 anterior teeth (six upper teeth and the other six lower teeth) and on the first permanent molars (two upper and two lower teeth). The presence of visible plaque was dichotomously logged per tooth (absence or presence of visible plaque). The total number of teeth with visible plaque was divided by the total number of teeth assessed and multiplied by 100, expressing the total value as a percentage of the visible plaque index. This value was categorized as ˂ 20% or ≥ 20%.^15^

The MIH assessment was conducted according to the criteria of the European Academy of Paediatric Dentistry (EAPD) for permanent teeth.^8^ MIH was classified as present when at least one permanent first molar was detected with a marked opacity greater than 2 mm in diameter, post‐eruptive enamel breakdown, atypical restorations with opacities at the margins, atypical caries lesions, or atypical extractions of the first permanent molars. Opacities restricted to permanent incisors without the involvement of molars were not considered MIH. They were classified as having MIH when at least one molar showed the signs. MIH severity was categorized into mild or severe.^16^ Teeth with opacities only were classified as mild, whereas post-eruptive breakdown or atypical caries/restoration were classified as severe.

Caries were detected using the criteria of the International Caries Detection and Assessment System II (ICDAS – II) along with the Activity Lesion Assessment (ALA), which evaluates visual characteristics, local susceptibility to plaque accumulation, and surface texture.^17-18^ The presence of moderate/severe active caries (ICDAS codes 3 to 6) was recorded: Localized degradation of the enamel (ICDAS code 3), shadow of underlying dentin (ICDAS code 4), distinct cavity in visible dentin (ICDAS code 5), extensive cavity in visible dentin (ICDAS code 6).^19^ The number of teeth with active dental caries was counted.

### Data analysis

Data were organized and analyzed in the Statistical Package for Social Science for Windows software, version 25.0 (IBM Corp., Armonk, N.Y., USA). Frequency analyses and the Kolmogorov-Smirnov normality test were conducted on numerical variables that did not follow a normal distribution. Bivariate analyses were conducted on the associations between the dependent variable (number of teeth with moderate/severe caries) and the independent variables (child’s sex, child’s age, caregiver’s education, family income, income dependents, sugar consumption, tooth brushing, visible plaque index, MIH) using the Mann-Whitney and Kruskal-Wallis tests. Poisson regression with robust variance was performed. The multivariate analysis was adjusted for variables in which p-values were less than 0.20, calculating the prevalence ratio (PR) and the 95% confidence interval (CI). Statistical significance was considered when p was less than 0.05.

## Results

Among the 430 children (accompanied by their parents/guardians) who were invited to participate in the study, 347 signed the informed consent form (response rate =80.70%). The final sample size included in the regression analysis resulted in a power of 75.83% to detect the difference between the mean number of carious teeth in the comparison groups. There were 175 girls and 172 boys, with a mean age of 8.63 years (SD=1.12).

The prevalence of moderate/extensive caries was 39.2%, and the mean number of teeth with moderate/extensive caries was 1.80 (SD±2.67). The mean number of decayed permanent teeth was 0.69 (SD=1.21), and the mean number of decayed primary teeth was 1.11 (SD=1.89). Among the children, 20.5% had at least one tooth with MIH. Specifically, 16 children (4.6%) had MIH in only one molar, 15 (4.3%) had MIH in two molars, 14 (4.0%) had MIH in three molars, and 26 (7.5%) had MIH in all four first permanent molars. In terms of severity, 11.8% of the children were classified as having mild MIH and 8.6% as severe MIH.

Table 1 shows the distribution of the mean number of teeth with moderate/extensive caries according to the independent variables. A significant association was observed between the number of teeth with extensive/moderate caries and the parents/guardian education, monthly family income, sugar consumption, visible plaque index, tooth brushing, and MIH (p<0.05).

The multivariate analysis (Table 2) demonstrated the number of teeth with moderate/extensive caries was associated with the presence of MIH in the child’s oral cavity (PR=1.45; 95% CI=1.03-2.04; p=0.031). Furthermore, the number of teeth with moderate/extensive caries was associated with a monthly family income of less than one minimum wage (PR=2.20; 95% CI=1.31-3.69; p=0.003), with sugar consumption (PR=1.82; 95% CI=1.32-2.51; p<0.001), and visible plaque index (PR=2.76; 95% CI=1.93-3.93; p<0.001).

**Table 2:** Poisson regression model for covariates associated with the number of teeth with moderate/extensive caries (n=347, Diamantina, Brazil).

Variables	Unadjusted PR (95% CI)	*p*	Adjusted PR	*p*
			**(95% CI)**	
**Sex of the child**				
Female	1			NS
Male	1.18 (0.86-1.61)	0.303	-	
**Child's age**				
7 years	1			NS
8 years	0.62 (0.28-1.40)	0.255		
9 years	1.24 (0.62-2.46)	0.538		
10 years	1.66 (0.85-3.26)	0.140	-	
**Caregiver's education**				
Higher education	1			NS
High school	1.84 (1.24-2.73)	**0.002**		
Basic education	1.92 (1.17-3.15)	**0.010**	-	
**Monthly family income**				
>3 minimum wages	1		1	
From 1 to 3 minimum wages	2.13 (1.27-3.56)	**0.004**	1.50 (0.90-2.51)	0.123
<1 minimum wage	3.38 (2.04-5.61)	**<0.001**	2.20 (1.31-3.69)	**0.003**
**Income dependents**				
≤3 people	1			NS
>3 people	1.18 (0.85-1.63)	0.331	-	
**Sugar consumption**				
<2 times a day	1		1	**<0.001**
≥2 times a day	2.34 (1.74-3.15)	**<0.001**	1.82 (1.32-2.51)	
**Tooth brushing**				
≥2 times a day	1			NS
<2 times a day	1.60 (1.06-2.41)	**0.026**	-	
**Visible Plaque Index**				
<20%	1		1	**<0.001**
≥20%	3.61 (2.54-5.11)	**<0.001**	2.76 (1.93-3.93)	
**MIH**				
Absence	1		1	**0.031**
Presence	1.60 (1.12-2.28)	**0.009**	1.45 (1.03-2.04)	

† PR: Prevalence ratio; ‡ CI: Confidence Interval; § NS: Not significant.

## Discussion

The findings demonstrated that children with MIH have a higher number of teeth with moderate/extensive caries. Moreover, children with high sugar consumption, high visible plaque index, and who belong to low-income families had more cases of carious teeth.

The prevalence of moderate/extensive caries was 39.2%, which is a considerably high value similar to previous studies that used similar criteria to evaluate the disease, with a variation from 32.4% to 43.5%.^20-21^ Despite significant advances in prevention, caries is still considered a public health problem due to its high prevalence and impact on individuals’ lives.^22-23^ This demonstrates that further efforts are needed to identify risk and protective factors for this disease, raise public awareness, and implement effective preventive health actions.

As to the prevalence of MIH, this study demonstrated that this change occurred in 20.5% of the evaluated children. This is slightly above the global prevalence (around 14.2%),^8^ however, it is like the prevalence of MIH found in different regions of Brazil, where MIH prevalence values vary from 18.4% to 20.4%.^24^ According to studies, prevalence values differ between countries, with regional differences within the same country.^25^

In this study, it was observed that children with MIH had a 1.45 times higher prevalence of teeth with carious lesions. Corroborating our findings, a systematic review demonstrated that a child with MIH is approximately 2.1 to 4.6 times more likely to have caries.^4^ The analysis was performed considering all teeth in the child’s oral cavity, since MIH can impact not only the directly affected teeth, but also the other teeth. This association can be explained by tooth sensitivity generally reported by patients with MIH, which can hinder brushing and flossing, resulting in bacterial plaque accumulation and increasing the risk of developing caries in both hypomineralized teeth and in other dental elements.^4,26^ Besides, post-eruptive disintegration can occur in teeth with MIH, which can cause discomfort and pain in children, leading to poor oral hygiene and favoring plaque accumulation.^4^ In fact, a high level of visible plaque is associated with dental caries development. This association is already consolidated and is justified by the fact that dental plaque provides a favorable environment for caries formation, as it harbors bacteria that produce harmful acids to teeth.^27^ Therefore, it is of paramount importance that once MIH is detected, preventative measures must be taken to prevent the accumulation of dental plaque, the development of carious lesions, and the reduction of MIH sensitivity and discomfort.

There is robust evidence demonstrating the association of low socioeconomic status with a greater risk of tooth decay in children.^28^ In this study, socioeconomic variables and variables regarding children’s habits were included in the regression model as confounding factors. It was observed that a monthly family income of less than one minimum wage was associated with a greater number of carious teeth. A systematic review that evaluated tooth decay and income in low- and middle-income countries found that families with low socioeconomic status had a 52% increased chance of their children developing caries.^3,29^ Another systematic review evaluated 164 articles worldwide, and it was observed that the prevalence of caries is higher in underdeveloped countries.^2^ Low income can hinder access to information as well as access to dental services, in addition to influencing behavioral and health habits.^30^

The caregiver’s education is also an important variable in assessing the families’ socioeconomic context. In this study, it was associated with dental caries in the unadjusted analysis and lost this association in the adjusted analysis due to collinearity with the income variable. Both caregiver’s education and income are factors that influence parents’ knowledge as well as their attitudes and practices, including their children’s eating and hygiene practices.^31^ Even when there is access to information, low levels of education can impair a person’s ability to process and understand preventive oral health information, as well as to seek treatment when necessary.^32^

The relationship between high sugar intake and caries is corroborated, widely recognized, and supported by several studies.^3,23,33,34^ Readily available sugars, such as those found in soft drinks, candies, and processed foods, have a significant role in caries progression. Such sugars are easily fermented by the bacteria in the plaque, resulting in the production of acids that demineralize tooth enamel.^33,34^ Furthermore, sticky foods, such as candies and chewing gum, stick to teeth for a long time, prolonging teeth exposure to sugars and increasing the risk of caries.^33,34^

Only active caries lesions were considered in this study, while the history of the disease was not evaluated via restorations or extracted teeth. This decision was made because active caries lesions represent the patient’s current risk, which was the focus of our assessment. Moreover, a previous study demonstrated that restorations in teeth with MIH can often be attributed to post-eruptive breakdown rather than caries lesions,^34^ which could lead to an overestimation of the disease prevalence.

The European Academy of Paediatric Dentistry (EAPD) recommends the age of eight years for the assessment of MIH. However, children aged from seven to ten years were included in this study to ensure a broader and more comprehensive evaluation. Studies suggest that the timing of permanent tooth eruption has changed in recent decades, with the emergence of permanent teeth occurring earlier over time.^35-37^ Such changes may be influenced by factors such as genetics, nutrition, and overall health.^38^ By the age of seven, most children have their first permanent molars and central incisors erupted, enabling an early assessment of MIH and its potential impacts.

Although this study provides relevant information, via cross-sectional studies, it is not possible to establish cause-and-effect relationships. The use of questionnaires to collect non-clinical data may result in response bias, as participants might provide inaccurate information or fail to recall past events accurately. Such biases can influence the results and affect data reliability. Complementing these studies with longitudinal research can provide a more comprehensive and robust view of the relationships between the variables of interest. Due to the vast Brazilian territorial extension, its large population, and significant demographic diversity, there is an essential need to document additional epidemiological studies to assess the prevalence of MIH throughout the country. This approach is crucial to understand the prevalence and geographic distribution of this dental condition in different regional contexts.

Since data collection was performed in a school environment, without the aid of an air jet to dry the teeth, it was decided to consider only ICDAS codes 3 to 6. Despite the possibility of identifying code 2 lesions, their activity could not be evaluated. Future studies conducted in clinical settings could incorporate early caries lesions in their analyses.

One of the strengths of this study was the population-based sample, instead of children who sought dental clinics, who may have greater treatment needs and increase selection bias. This study also addressed other known risk factors for caries, such as frequency of sugar intake and oral hygiene standards. The control of these variables was indicated by previous studies and is crucial in determining the causal factors of dental caries given their multifactorial nature (3). The use of well-established criteria for diagnosing oral conditions, such as ICDAS and EAPD, must be highlighted. The findings of this study are relevant in guiding personalized clinical behaviors and contribute to the development of preventive public interventions in oral health.

## Conclusion

This study underscores the significant association between MIH and a higher number of teeth with moderate/extensive caries in children. Such findings highlight the need for targeted preventive measures and individualized clinical approaches to address the oral health challenges posed by MIH. The study also reaffirms the critical roles of socioeconomic status, dietary habits, and oral hygiene in the prevalence of dental caries. Children from low-income families, those who have high sugar consumption, and those who have a high visible plaque index were found to be at greater risk for carious lesions.
